# Acute *In Vivo* Response to an Alternative Implant for Urogynecology

**DOI:** 10.1155/2014/853610

**Published:** 2014-07-17

**Authors:** Sabiniano Roman Regueros, Maarten Albersen, Stefano Manodoro, Silvia Zia, Nadir I. Osman, Anthony J. Bullock, Christopher R. Chapple, Jan Deprest, Sheila MacNeil

**Affiliations:** ^1^Department of Material Science and Engineering, Kroto Research Institute, University of Sheffield, Sheffield S3 7HQ, UK; ^2^Department of Development and Regeneration, University Hospital Gasthuisberg, Catholic University of Leuven, 3000 Leuven, Belgium; ^3^Centre for Surgical Technologies, Catholic University of Leuven, 3000 Leuven, Belgium; ^4^Royal Hallamshire Hospital, Sheffield S10 2JF, UK

## Abstract

*Purpose*. To investigate *in vivo* the acute host response to an alternative implant designed for the treatment of stress urinary incontinence (SUI) and pelvic organ prolapse (POP). *Methods*. A biodegradable scaffold was produced from poly-L-lactic acid (PLA) using the electrospinning technique. Human and rat adipose-derived stem cells (ADSCs) were isolated and characterized by fluorescence-activated cell sorting and differentiation assays. PLA scaffolds were seeded and cultured for 2 weeks with human or rat ADSCs. Scaffolds with and without human or rat ADSCs were implanted subcutaneously on the abdominal wall of rats. After 3 and 7 days, 6 animals from each group were sacrificed. Sections from each sample were analyzed by Haematoxylin and Eosin staining, Sirius red staining, and immunohistochemistry for CD68, PECAM-1, and collagen I and III. *Results*. Animals responded to the scaffolds with an acute macrophage response. After 7 days of implantation, there was extensive host cell penetration, new blood vessel formation, and new collagen deposition throughout the full thickness of the samples without obvious differences between cell-containing and cell-free scaffolds. *Conclusions*. The acute *in vivo* response to an alternative implant (both with and without cells) for the treatment of SUI and POP showed good acute integration into the host tissues.

## 1. Introduction

Surgical implantation of both natural and synthetic cell-free materials is the current standard of care in many parts of the world in the treatment of stress urinary incontinence (SUI) and pelvic organ prolapse (POP) [1]. Autologous fascia, long used as a sling material for SUI, requires specialized training and is limited by the amount that can be harvested with associated donor site morbidity [2]. Nondegradable polypropylene synthetic meshes, introduced as a less invasive alternative, have been widely used over the past decade; nevertheless, increasing reports of serious complications with these materials such as vaginal or urinary tract exposure, chronic pain, and voiding dysfunction are now emerging [2–4].

Although many factors may influence the outcome of mesh surgery, including physical properties of the material and surgical and constitutional factors [1], the host response is particularly important. Nondegradable polypropylene implants cannot be remodelled and induce release of cytokines, and some patients respond to them with chronic inflammation followed by an unsuitable fibrosis which can lead to the above complications [5]. Alternatively, the outcomes of using degradable biological grafts, trialled in limited clinical studies, are mixed. Animal collagen grafts have been found to fail due to quick degradation and while chemical cross-linking overcomes this it can result in poor graft integration [6].

We have previously shown the potential of poly-lactic acid (PLA), an FDA approved polymer synthesized into a microfiber scaffold, to develop* in vitro* into an engineered tissue when seeded with adipose derived stem cells (ADSCs) producing the key extracellular matrix (ECM) proteins [7]. In addition, we also showed* in vitro* that PLA scaffolds are more biocompatible than polypropylene meshes with mechanical properties close to those of native tissues [8].

Therefore, we aim to develop an alternative material for the treatment of SUI and POP which degrades slowly while the introduction of autologous cells to these scaffolds will produce new ECM. We hypothesise that the absorbable material is less likely to result in exposure through vaginal tissues and the cellular component will encourage tissue regeneration and good integration in the host tissues leading to better outcomes than current materials used to treat SUI and POP.

Since the acute host response elicited by any biomaterial is critical to its integration into the host tissues [9], in this study, we sought to assess this response in animals by comparing PLA scaffolds implanted with and without human ADSCs. Rat ADSCs were also included in this study as an allogeneic implantation control.

## 2. Materials and Methods

Scaffold production and human ADSCs isolation were performed in the Kroto Research Institute, University of Sheffield. Cells and PLA scaffolds were sent to the Laboratory of Experimental Gynaecology, University Hospital Leuven, for sample preparation. Rat ADCSs isolation and characterization of rat and human ADSCs were also carried out in this laboratory. Animal surgery was conducted in the Centre for Surgical Technologies, Katholieke Universiteit Leuven. After the sacrifice, samples were paraffin fixed in the Laboratory of Experimental Gynecology and histological analysis was conducted at the Kroto Research Institute.

### 2.1. PLA Scaffold Synthesis

A 10% PLA solution (Sigma-Aldrich, Dorset, UK) dissolved in dichloromethane was made (w/v). PLA scaffolds were produced aseptically by electrospinning as previously described [7]. Thereafter scaffolds were heat-annealed in a dry oven at 60°C for 3 hours.

### 2.2. ADSCs Isolation and Culture

ADSCs were sourced from human subcutaneous fat donated on an anonymous basis under a research tissue bank licence (number 08/H1308/39) under the Human Tissue Authority. Isolation and culture were performed as previously described from 10 mL of fat tissue [7]. Cells at passage 4 were cryopreserved in 1 mL of 10% DMSO (dimethyl sulfoxide) in fetal calf serum (FCS) (Advanced Protein Products, Brierley Hill, UK). Once in Leuven, cells were resurrected and then maintained at 37°C and 5% CO_2_ with Dulbecco's Modified Eagle's Medium (DMEM) supplemented with 10% FCS, 1% penicillin/streptomycin, 1% glutamine, and 0.25% fungizone (Gibco Invitrogen, Paisley, UK) (all experiments were in DMEM medium plus 10% FBS unless stated otherwise). Cells were used at passage 6 in experiments.

All animal procedures were approved by the ethical committee of the Katholieke Universiteit Leuven with the project number P163_2011.

After isoflurane anaesthesia, Sprague-Dawley rats were sacrificed by cervical dislocation. After laparotomy, subcutaneous fat was processed to isolate rat ADSCs following the above human ADSCs isolation protocol. Cells at passage 4 were used in experiments.

### 2.3. Fluorescence-Activated Cell Sorting (FACS)

Human ADSCs were characterized using flow cytometry analysis [10]. 100,000 cells were harvested and incubated with either FITC or PE-conjugated antibodies against human CD24, CD90, CD44, CD105, CD73, HLA-ABC, HLA-DR, CD34, and CD45 (BD Bioscience, Erembodegem, Belgium) and CD29 (Acris, Herford, Germany) mouse anti-human monoclonal antibodies and appropriate isotype controls. Stained cells were analyzed using a Beckton Dickinson flow cytometer (Beckton Dickinson, Franklin Lakes, NJ, USA) using the Cell Quest software and data were analysed using the FlowJo software (Tree Star, Ashland, OR, USA).

The same analysis was performed for rat ADSCs using either FITC or PE-conjugated antibodies against rat I-E[*κ*] CD90, CD44, CD31, CD45, and CD11b (BD Bioscience) and CD29 (Acris) mouse anti-rat monoclonal antibodies and appropriate isotype controls.

### 2.4. Differentiation Assays

The multipotency potential of human and rat ADSCs was evaluated by differentiation assays as previously described [11].

After 3 weeks in culture with osteogenic or adipogenic medium, cells were fixed and stained by incubation for 30 minutes with 1 mg/mL Alizarin Red solution (Sigma-Aldrich) or filtered 0.3% Oil Red O (Sigma-Aldrich) in 60% isopropanol (Fisher Scientific, UK Ltd.) (w/v), respectively.

### 2.5. Scaffold Preparation and Cell Seeding

Sterile PLA scaffolds of 1.5 × 1.5 cm were seeded with 500,000 human or rat ADSCs using steel rings as a seeding well of 1 cm diameter. All samples were cultured in DMEM medium at 37°C in a 5% CO_2_ atmosphere. We also included cell-free scaffolds in medium as controls.

### 2.6. Implantation

After 2 weeks in culture, 3 groups of samples were implanted—plain PLA scaffolds and PLA scaffolds cultured with human or rat ADSCs. Only one sample was implanted in every female Sprague-Dawley female rat with 12 rats per each of the 3 groups (36 rats in total).

**Figure 1:**
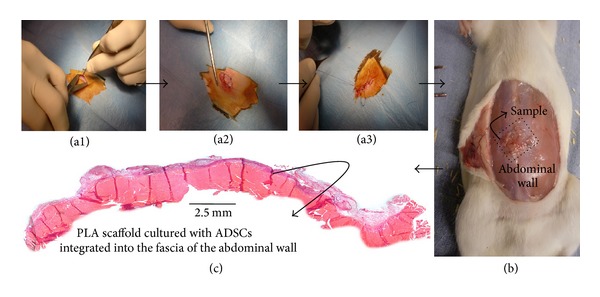
Animal surgical procedure. (a1) Skin incision and flaps of subcutaneous layer were raised from the top of the abdominal wall. (a2) Suture of sample at four corners. (a3) Subcutaneous and skin layers closure. (b) Animals sacrificed and appearance of sample on top of abdominal wall. (c) Representative light microscopy H&E stained panoramic image of the abdominal wall of female Sprague-Dawley rat, after 3 days of implantation of PLA scaffold on top, previously cultured with human ADSCs in DMEM medium for 2 weeks.

Animals were placed in 100% isoflurane (Isoba) and kept under isoflurane anaesthesia via a nose cone. After the belly of the animal was shaved and disinfected, the abdominal skin was incised and flaps of the subcutaneous layer were raised (Figure 1). Samples were sutured on the abdominal wall with nonabsorbable sutures (Prolene* (4-0/RB-1 17 mm 1/2c; Ethicon, Groot Bijgaarden, Belgium)) on each corner. Subcutaneous layer and skin were closed with resorbable sutures (Vicryl* (2-0/FS-1 24 mm 3/8c, Ethicon)). Animals were weaned from anaesthesia and observed for recovery.

### 2.7. Sacrifice and Sample Fixation

At 3 and 7 days after implantation, 6 animals from each group were sacrificed by intracardiac injection of T-61 (embutramide 200 mg, mebenzonium iodide 50 mg, and tetracaine hydrochloride 5 mg, per mL) (Intervet, International B.V.). Abdominal wall pieces of 2 cm^2^ containing implants on top were explanted (Figure 1). Samples were fixed in 10% neutral buffered formalin and paraffin embedded (Chandon CITADEL 1000, HVL).

### 2.8. Histology

Sections 6 *μ*m thick were cut from the paraffin embedded samples with a microtome (Leica TP 1020 Automatic Tissue Processor) and placed on Superfrost plus slides (Menzel-Gläser, Denmark).

Conventional Haematoxylin and Eosin (H&E) staining was performed as previously described [12]. Slides were then mounted in DPX mounting medium (Fisher Scientific) with a coverslip.

For immunohistochemistry procedure sections were rehydrated, then delineated with a Dako pen, and treated with 0.05% trypsin (Sigma-Aldrich) for 20 minutes at 37°C. The samples were blocked using donkey serum (ImmunoCruz goat ABC Staining System, Santa Cruz Biotechnology, Inc.) for 1 hour. Sections were incubated with one of four monoclonal antibodies overnight: mouse anti-rat CD68 (1 : 200; Abcam, UK), goat anti-rat PECAM-1 (1 : 50; Santa Cruz Biotechnology, Inc.), goat anti-human collagen I (AbD Serotec, Oxford, UK), and goat anti-human collagen III (AbD Serotec, Oxford, UK). This was followed by 1 hour incubation with secondary antibodies: biotinylated goat anti-mouse Ig (1 : 200; BD, Pharmingen) and biotinylated anti-goat Ig (1 : 200; ImmunoCruz goat ABC Staining System, Santa Cruz Biotechnology, Inc.). After incubation with an avidin and biotinylated horseradish peroxidase, the target proteins were visualized by incubation in peroxidase substrate and DAB chromogen (ImmunoCruz goat ABC Staining System). Samples were then counterstained with Haematoxylin, dehydrated, and mounted as per H&E protocol.

Three groups of controls were performed—samples incubated without primary and secondary antibodies, or incubated only with secondary antibodies. Semiquantitative assessment of the extent of immunostaining was done on a blinded observer basis using a qualitative grading scale; absent = 0, mild presence = 1, large presence = 2, abundance = 3, and great abundance = 4. Example photographs depicting 0, 1, 2, 3, and 4 were provided for reference and the median value from these scores was used [7].

For total collagen staining, sections were rehydrated, following the same protocol as for H&E, and then incubated with Sirius red (0.1% w/v Direct Red 80 in saturated picric acid, Sigma-Aldrich) for 1 hour. Samples were then rinsed briefly in distilled water and washed in acidified water (0.5% acetic acid, VWR International Ltd.) for 1 minute. Finally samples were dehydrated and mounted as per the H&E protocol.

### 2.9. Statistics

Differences for the semiquantitative assessment of the extent of immunostaining were statistically tested against a null hypothesis of no difference between samples using a two-sample Student's *t*-test with equal variance not assumed (significance = *P* < 0.05).

## 3. Results

**Figure 2:**
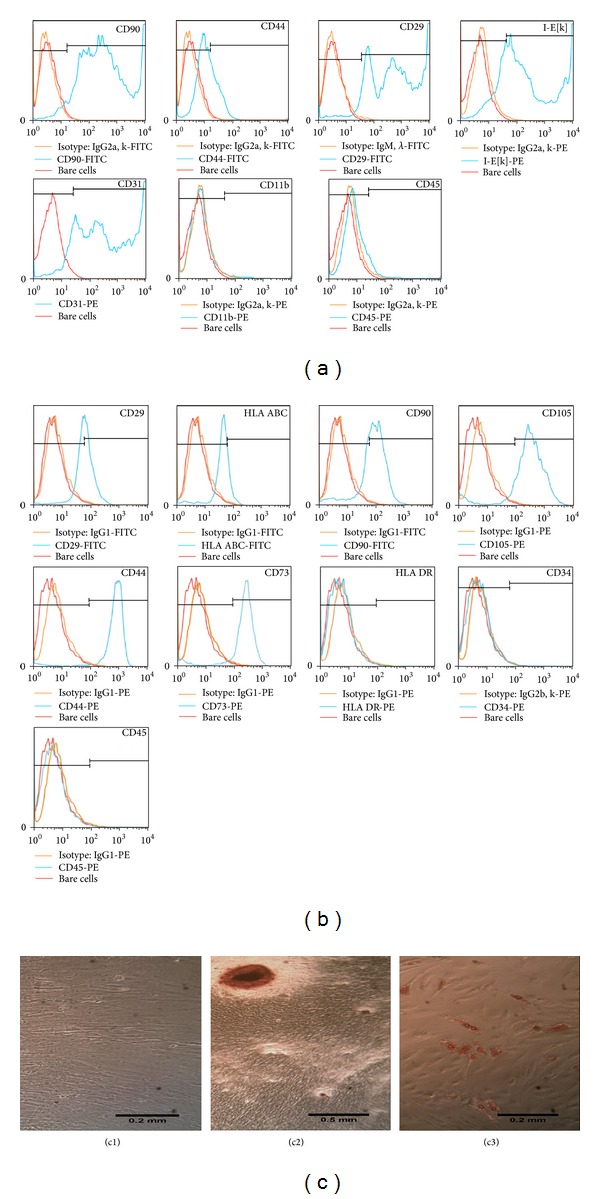
Characterization of ADSCs. Rat (a) and human (b) ADSCs isolated from subcutaneous adipose tissue characterized by FACS showing fluorescent intensity for bare cells in red colour, for isotypes controls in orange colour, and for each specific antigen marker in blue colour. At the bottom, differentiation assays showing potential for osteogenic (c2) and adipogenic (c3) lineages, preceded by human ADSCs cultured in DMEM medium as control (c1).

Human and rat ADSCs were positively and negatively characterized by expression of specific cell surface antigens, as previously described [10], and by their differentiation potential (Figure 2).

All animals survived both the operation and the period of implantation without any observed alteration in their physiological functions. No signs of infection were observed when harvesting the samples and all of them (PLA scaffolds previously cultured with and without cells) were identified on the subcutaneous fascia which covers the abdominal wall muscles (Figure 1).

**Figure 3:**
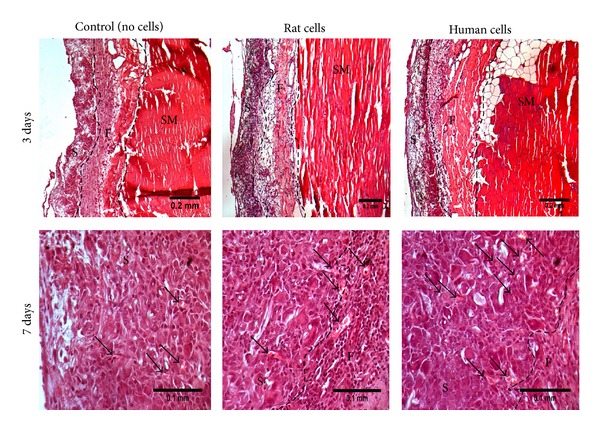
Morphological appearance of the implanted samples. Representative light microscopy H&E stained sections of abdominal wall of female Sprague-Dawley rat after 3 and 7 days of implantation of PLA scaffold on top, previously cultured with and without (control) rat or human ADSCs in DMEM medium for 2 weeks. At 7 days, all samples presenting several small blood vessels are identified by (↑). Scale bars of 0.2 mm for images from 3 days implantation and 0.1 mm for images from 7 days implantation. (S) Sample; (F) Fascia; and (SM) Skeletal Muscle.

After 3 days of implantation, host cells infiltrated samples, as seen in samples implanted without cells for H&E staining. After 7 days the cell infiltration was increased in all samples, and new small blood vessels were visible inside the samples (Figure 3).

**Figure 4:**
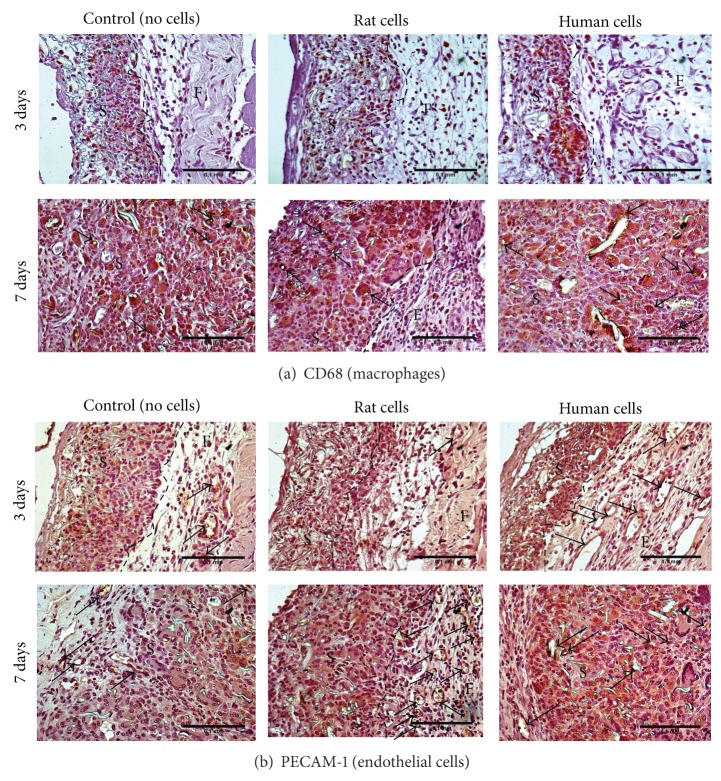
Assessment of the acute host response against the implanted samples. Representative light microscopy image of sections of abdominal wall of female Sprague-Dawley rats after 3 and 7 days of implantation of PLA scaffold on top, previously cultured with and without (control) rat or human ADSCs in DMEM medium for 2 weeks; following immunohistochemistry for anti-CD68 antibody (a) or anti-PECAM-1 antibody (b). (a) Macrophages surrounding individual PLA fibres are identified by (↑). (b) Endothelial cells stained for PECAM-1 around blood vessels are identified by (↑). Scale bars of 0.1 mm. (S) Sample; (F) Fascia.

**Figure 5:**
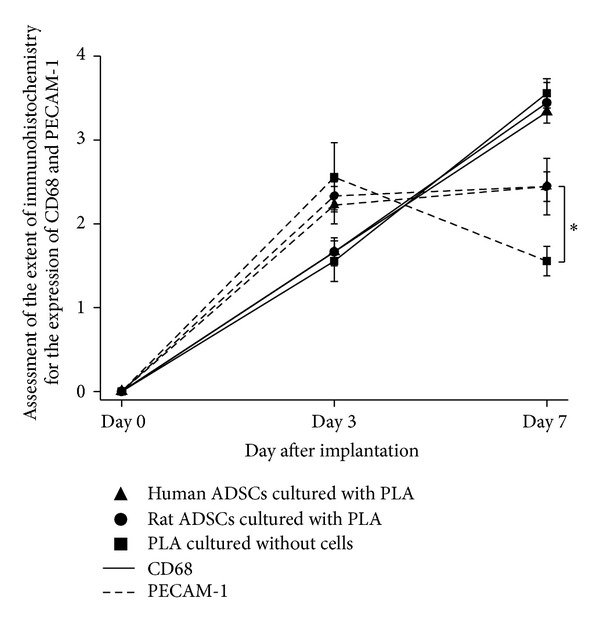
Semiquantitative analyses of the host response against the implanted samples. Assessment of the extent of immunohistochemistry using a blind scoring for the expression of CD68 and PECAM-1 from sections of abdominal wall of female Sprague-Dawley rat after 3 and 7 days of implantation of PLA scaffold on top, previously cultured with and without (control) rat or human ADSCs in DMEM medium for 2 weeks. Results shown as mean ± SEM (*n* = 6). Scale: 0 = absent, 1 = mild presence, 2 = large presence, 3 = abundance, and 4 = great abundance.

At day 3, CD68 positive cells were seen throughout the samples, localized inside them and not found in the surrounding tissues (Figure 4). Semiquantitative assessment of the immunohistochemistry demonstrated this staining to be moderate, becoming more intense after 7 days implantation (Figure 5).

A moderate PECAM-1 staining was identified after 3 days with no differences between groups. By 7 days, there was a similar expression between samples with rat or human ADSCs but statistically lower staining for cell-free samples (Figure 5). Although PECAM-1 stained many cells inside the samples, similarly to CD68, this was also identified around large blood vessels in the abdominal fascia at day 3; while, after 7 days of implantation, new small blood vessels inside all samples were stained (Figure 4).

**Figure 6:**
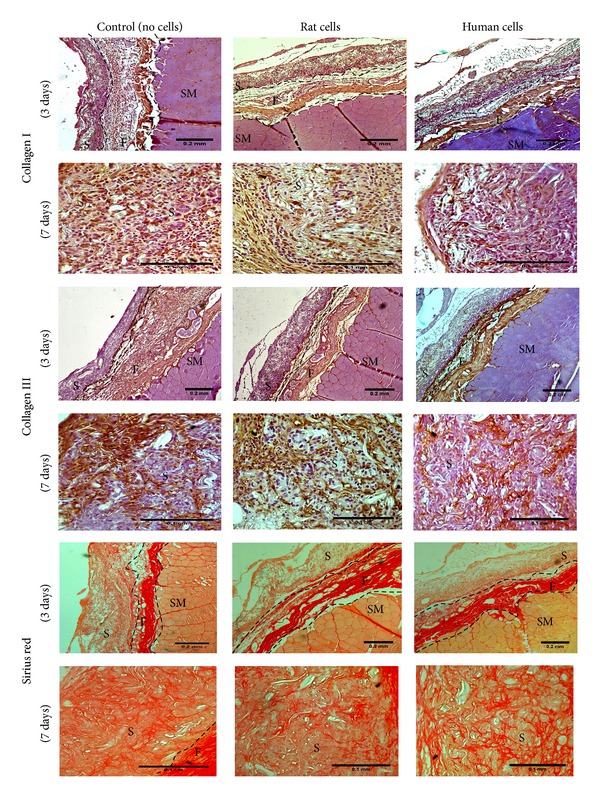
Assessment of new extracellular matrix formation in the samples implanted. Representative light microscopy of sections of abdominal wall of female Sprague-Dawley rat after 3 days of implantation of PLA scaffold on top, previously cultured with and without (control) rat or human ADSCs in DMEM medium for 2 weeks; following immunohistochemistry for anti-collagen I and anti-collagen III antibodies, or Sirius red staining. Scale bars of 0.2 mm for images from 3 days implantation and 0.1 mm for images from 7 days implantation. (S) Sample; (F) Fascia; and (SM) Skeletal Muscle.

After 3 days of implantation, immunohistochemistry for collagen III and Sirius red staining for total collagen revealed a thin layer of collagen production on the lower surface of all the samples, and at day 7, thin new collagen fibres were visible throughout the samples (Figure 6). Collagen I staining at day 3 was minimally found around cells inside samples and only for samples implanted with human or rat cells; although, after 7 days, this minimal staining was found inside all samples (Figure 6).

## 4. Discussion

Since the U.S. Food and Drug Administration announced serious complications with current surgical meshes used to treat POP and SUI [13], several studies have investigated the host response in animals to different cell-free synthetic and biological materials. This is viewed as a critical indicator in predicting their long-term outcomes [14–16].

Many animal studies show that polypropylene meshes provoke a fairly pronounced inflammation leading to a massive cell infiltration into the scaffold and ultimately to new collagen production described as a vigorous fibrotic process [17–19]. These studies also reported an increase in the stiffness of polypropylene after its implantation due to this fibrosis. Some fibrosis may be desirable for successful outcomes when treating SUI or POP. This is an area where it is currently difficult to obtain data correlating patient's responses to clinical outcome. Alternatively, irreversible plastic deformation of this material may explain why it could “cheesewire” through the patient's tissues leading to exposure in some patients.

To improve integration into native tissues, a few groups have investigated* in vitro* and in hernia repair animal models light polypropylene meshes which have been modified by surface coating with collagen, titanium, or absorbable polymers. While some of these animal studies found higher biocompatibility for the polypropylene light meshes compared to the polypropylene control group [20, 21], others found that the outcomes were very similar between the two groups [22]. Some of these meshes have now been introduced into the market since these are thought to be associated with lower complications. However, a review of randomized controlled trials using these meshes for human hernia repair found higher recurrence rates compared to conventional polypropylene meshes [23].

On the other hand, the tissue engineering field has recently introduced new biomaterials which can be used for several clinical applications. Neural stem cells or osteoblasts have been combined with electrospun PLA scaffolds with potential for peripheral nerve repair [24] and as a bone substitute [25], respectively.

In addition, PLA monofilament meshes have been assessed* in vivo* with an incisional hernia Wistar rat model used to simulate vaginal wall repair [26]. Compared to polypropylene, the PLA scaffold retained an acceptable strength 8 months after implantation, showed a significantly lower inflammatory response, and the collagen produced was better organized. The same authors also reported PLA meshes to have less infection risk compared to other meshes in a rat infected abdominal model [27].

Similarly to our study, only two research groups have previously assessed in animals an engineered tissue for the treatment of SUI and POP which were developed from biodegradable polyglycolic acid (PGA) [28] or poly-lactic-glycolic acid (PLGA) [13] scaffolds. Both studies found good integration into host tissues with neofascia formation. However, the rate of degradation of scaffolds* in vivo* is rapid (within weeks) for PGA and proportionally slower as PLA, which is much slower to degrade, is added to the polymer solution. Our group has shown* in vivo* that electrospun scaffolds of 50% PGA and 50% PLA are degraded within 8 weeks in rats and PLGA (75/25) scaffolds last for more than 3 months whilst PLA scaffolds are present after 12 months of implantation [12]. Thus the rate of breakdown is tunable and predictable and has relevance to maintenance of mechanical properties of the implants.

The current study also aimed to explore the acute response to the use of mesenchymal stem cells which have been already used in women [29] to treat SUI by cell injection into the urethral sphincter and submucosa.

Large numbers of these cells can be quickly isolated using a minimally invasive liposuction in humans [30]. ADSCs do not differentiate when cultured in basic DMEM medium, displaying fibroblastic behavior and producing an endogenous ECM [31]; in addition to this, they have been shown to release a growth factor to stimulate fibroblast proliferation with the potential to regenerate connective tissues [32]. Furthermore, ADSCs have the potential to inhibit inflammatory responses by secretion of the inhibitor of tumor necrosis factor *α* [32] and a subpopulation of ADSCs expresses an endothelial surface antigen (CD34) which can promote neovascularization [33].

In our study we implanted human cells in immunocompetent Sprague-Dawley rats; however, rat cells were included as a control. All samples were implanted in different rats since interpretation of responses to different materials in the same animal is not recommended with a body wide immune response.

ADSCs were well characterized prior to implantation but they were not tracked post implantation so it is not possible to comment on any direct regenerative effect of these cells. Alternatively, after few days implantation the major aspect to assess was the host inflammatory response elicited against these implants and, actually, this was very similar for cell-free scaffolds and those seeded with human or rat ADSCs.

All PLA scaffolds, both without and with cells, were integrated into the fascia of the abdominal wall with rapid host cell infiltration and ingrowth of small blood vessels.

The macrophage response against all samples was evident, particularly 7 days after implantation as identified by CD68+ cells [9]. This response seems to be specific to the synthetic foreign material since macrophages were not found in tissues surrounding the samples and macrophages enclosed individual PLA fibres (Figure 4).

Although PECAM-1 is expressed on platelets and subsets of leukocytes, it mainly stains endothelial cells with cell adhesion, transendothelial migration of myeloid-derived cells, and angiogenesis functions; and therefore, it has been widely used to assess neovascularization [9]. Since PECAM-1 staining was higher at day 7 for cell-seeded samples compared to samples implanted without cells, this could be interpreted as more myeloid-derived cell infiltrates and/or higher neovascularization as identified by small blood vessels inside cell-seeded samples.

Macroporous polypropylene mesh is said to be more favourable to permit host cell infiltration [2]. The current study shows that a microporous electrospun PLA scaffold permitted the infiltration of macrophages throughout its entire thickness which means that this scaffold is no barrier to macrophage activity so their ability to tackle bacterial infection would not be compromised. Additionally, the host cell infiltration led to ECM formation, as seen particularly for collagen III, which is indicative of remodelling of the implant leading to good integration into host tissues [9]. Alternatively, collagen I was minimally detected in all samples and, at day 3, it was only found in samples implanted with human or rat cells which may suggest production of this by the cells during the initial period of their culture* in vitro* [7].

In animal studies, the host response to materials used to treat SUI and POP is often analyzed after 7, 30, and 90 days after implantation. While acute inflammatory responses and integration into native tissues, including neovascularization and ECM production, can be assessed in the short-term by subcutaneous implantation in rats [34]; long-term implantation (30 and 90 days) usually in larger animals also allows the evaluation of chronic immune responses and the evaluation of any changes in mechanical properties after implantation [17–19].

Therefore, the major limitations of this work are its short-term nature since this model cannot be used to assess whether a chronic immune response ensues or the regenerative/angiogenesis potential of the cell-seeded scaffolds. Additionally, implanted ADSCs were not labelled, something that will be necessary in longer-term experiments to provide information on their survival or migration.

## 5. Conclusion

For all groups, an alternative implant designed for urogynecology showed host cell infiltration, mainly due to a macrophage response against the foreign material as a normal wound healing mechanism, which led to neotissue production with new blood vessels formation—all early indicators of constructive remodelling for long-term integration into host tissues [9].

Our future experiments will now progress to a longer term (3 months) rabbit fascial-defect model to investigate the development of any chronic immune response, the fate of the ADSCs, and, very crucial, the biomechanical properties of the implant after several months of implantation. Ultimately, our ideal approach to achieve an economical final clinical product would be to combine these scaffolds with patient's cells just before being surgically implanted on the same operation as rapid extraction of ADSCs from fresh lipoaspirate is being developed currently [35].

## References

[1] Chapple CR, Raz S, Brubaker L, Zimmern PE (2013). Mesh sling in an era of uncertainty: lessons learned and the way forward. *European Urology*.

[2] Birch C (2005). The use of prosthetics in pelvic reconstructive surgery. *Best Practice and Research: Clinical Obstetrics and Gynaecology*.

[3] Bako A, Dhar R (2009). Review of synthetic mesh-related complications in pelvic floor reconstructive surgery. *International Urogynecology Journal*.

[4] Jones R, Abrams P, Hilton P, Ward K, Drake M (2010). Risk of tape-related complications after TVT is at least 4%. *Neurourology and Urodynamics*.

[5] Zheng F, Xu L, Verbiest L, Verbeken E, De Ridder D, Deprest J (2007). Cytokine production following experimental implantation of xenogenic dermal collagen and polypropylene grafts in mice. *Neurourology and Urodynamics*.

[6] Davila GW, Drutz H, Deprest J (2006). Clinical implications of the biology of grafts: conclusions of the 2005 IUGA grafts roundtable. *International Urogynecology Journal and Pelvic Floor Dysfunction*.

[7] Roman S, Mangera A, Osman NI, Bullock AJ, Chapple CR, MacNeil S (2013). Developing a tissue engineered repair material for treatment of stress urinary incontinence and pelvic organ prolapse-which cell source?. *Neurourology and Urodynamics*.

[8] Mangera A, Bullock AJ, Roman S, Chapple CR, Macneil S (2013). Comparison of candidate scaffolds for tissue engineering for stress urinary incontinence and pelvic organ prolapse repair. *British Journal of Urology*.

[9] Ulrich D, Edwards SL, White JF (2012). A preclinical evaluation of alternative synthetic biomaterials for fascial defect repair using a rat abdominal hernia model. *PLoS ONE*.

[10] Zia S, Toelen J, da Cunha MM, Dekoninck P, de Coppi P, Deprest J (2013). Routine clonal expansion of mesenchymal stem cells derived from amniotic fluid for perinatal applications. *Prenatal Diagnosis*.

[11] Kaewkhaw R, Scutt AM, Haycock JW (2011). Anatomical site influences the differentiation of adipose-derived stem cells for Schwann-cell phenotype and function. *GLIA*.

[12] Blackwood KA, McKean R, Canton I (2008). Development of biodegradable electrospun scaffolds for dermal replacement. *Biomaterials*.

[13] Hung MJ, Wen MC, Hung CN, Ho ES, Chen GD, Yang VC (2010). Tissue-engineered fascia from vaginal fibroblasts for patients needing reconstructive pelvic surgery. *International Urogynecology Journal*.

[14] Konstantinovic ML, Ozog Y, Spelzini F, Pottier C, de Ridder D, Deprest J (2010). Biomechanical findings in rats undergoing fascial reconstruction with graft materials suggested as an alternative to polypropylene. *Neurourology and Urodynamics*.

[15] Ozog Y, Konstantinovic M, Zheng F (2009). Porous acellular porcine dermal collagen implants to repair fascial defects in a rat model: Biomechanical evaluation up to 180 days. *Gynecologic and Obstetric Investigation*.

[16] Claerhout F, Verbist G, Verbeken E, Konstantinovic M, de Ridder D, Deprest J (2008). Fate of collagen-based implants used in pelvic floor surgery: a 2-year follow-up study in a rabbit model. *The American Journal of Obstetrics and Gynecology*.

[17] Hilger WS, Walter A, Zobitz ME, Leslie KO, Magtibay P, Cornella J (2006). Histological and biomechanical evaluation of implanted graft materials in a rabbit vaginal and abdominal model. *The American Journal of Obstetrics and Gynecology*.

[18] Pierce LM, Grunlan MA, Hou Y, Baumann SS, Kuehl TJ, Muir TW (2009). Biomechanical properties of synthetic and biologic graft materials following long-term implantation in the rabbit abdomen and vagina. *The American Journal of Obstetrics and Gynecology*.

[19] Dora CD, Dimarco DS, Zobitz ME, Elliott DS (2004). Time dependent variations in biomechanical properties of cadaveric fascia, porcine dermis, porcine small intestine submucosa, polypropylene mesh and autologous fascia in the rabbit model: implications for sling surgery. *Journal of Urology*.

[20] Böhm G, Ushakova Y, Alizai HP (2011). Biocompatibility of PLGA/sP(EO-stat-PO)-coated mesh surfaces under constant shearing stress. *European Surgical Research*.

[21] Lukasiewicz A, Skopinska-Wisniewska J, Marszalek A, Molski S, Drewa T (2013). Collagen/polypropylene composite mesh biocompatibility in abdominal wall reconstruction. *Plastic & Reconstructive Surgery*.

[22] Junge K, Rosch R, Klinge U (2005). Titanium coating of a polypropylene mesh for hernia repair: effect on biocompatibilty. *Hernia*.

[23] Weyhe D, Belyaev O, Müller C (2007). Improving outcomes in hernia repair by the use of light meshes—a comparison of different implant constructions based on a critical appraisal of the literature. *World Journal of Surgery*.

[24] Murray-Dunning C, McArthur SL, Sun T, McKean R, Ryan AJ, Haycock JW (2011). Three-dimensional alignment of schwann cells using hydrolysable microfiber scaffolds: strategies for peripheral nerve repair. *Methods in Molecular Biology*.

[25] Paletta JRJ, Mack F, Schenderlein H (2011). Incorporation of osteoblasts (MG63) into 3D nanofibre matrices by simultaneous electrospinning and spraying in bone tissue engineering. *European Cells and Materials*.

[26] De Tayrac R, Chentouf S, Garreau H (2008). In vitro degradation and in vivo biocompatibility of poly(lactic acid) mesh for soft tissue reinforcement in vaginal surgery. *Journal of Biomedical Materials Research B Applied Biomaterials*.

[27] Mathé M-L, Lavigne J-P, Oliva-Lauraire M-C, Guiraud I, Marès P, de Tayrac R (2007). Comparison of different biomaterials for vaginal surgery using an in vivo model of meshes infection in rats. *Gynecologie Obstetrique Fertilite*.

[28] de Filippo RE, Yoo JJ, Atala A (2003). Engineering of vaginal tissue in vivo. *Tissue Engineering*.

[29] Carr LK, Steele D, Steele S (2008). 1-year follow-up of autologous muscle-derived stem cell injection pilot study to treat stress urinary incontinence. *International Urogynecology Journal*.

[30] Roche R, Festy F, Fritel X (2010). Stem cells for stress urinary incontinence: the adipose promise. *Journal of Cellular and Molecular Medicine*.

[31] Kang JW, Kang K, Koo HC, Park JR, Choi EW, Park YH (2008). Soluble factors-mediated immunomodulatory effects of canine adipose tissue-derived mesenchymal stem cells. *Stem Cells and Development*.

[32] Vermette M, Trottier V, Ménard V, Saint-Pierre L, Roy A, Fradette J (2007). Production of a new tissue-engineered adipose substitute from human adipose-derived stromal cells. *Biomaterials*.

[33] Traktuev DO, Merfeld-Clauss S, Li J (2008). A population of multipotent CD34-positive adipose stromal cells share pericyte and mesenchymal surface markers, reside in a periendothelial location, and stabilize endothelial networks. *Circulation Research*.

[34] Thiel M, Rodrigues Palma PC, Riccetto CLZ, Dambros M, Netto NR (2005). A stereological analysis of fibrosis and inflammatory reaction induced by four different synthetic slings. * British Journal of Urology*.

[35] Bosio A, Huppert V, Donath S, Hennemann P, Malchow M, Heinlein UAO (2009). Isolation and enrichment of stem cells. *Advances in Biochemical Engineering/Biotechnology*.

